# Tea (*Camellia sinensis*) Ameliorates Hyperuricemia via Uric Acid Metabolic Pathways and Gut Microbiota

**DOI:** 10.3390/nu14132666

**Published:** 2022-06-27

**Authors:** Dan Wu, Ruohong Chen, Qiuhua Li, Xingfei Lai, Lingli Sun, Zhenbiao Zhang, Shuai Wen, Shili Sun, Fanrong Cao

**Affiliations:** 1 College of Horticulture, South China Agricultural University, Guangzhou 510640, China; wudan@stu.scau.edu.cn; 2 Tea Research Institute, Guangdong Academy of Agricultural Sciences/Guangdong Provincial Key Laboratory of Tea Plant Resources Innovation & Utilization, Guangzhou 510640, China; chenruohong@tea.gdaas.cn (R.C.); liqiuhua@tea.gdaas.cn (Q.L.); laixingfei@tea.gdaas.cn (X.L.); sunlingli@tea.gdaas.cn (L.S.); zhangzhenbiao@tea.gdaas.cn (Z.Z.); wenshuai@tea.gdaas.cn (S.W.)

**Keywords:** *Camellia sinensis*, six types of tea, hyperuricemia, uric acid metabolism disorder, oxidative stress, gut microbiota

## Abstract

Hyperuricemia (HUA) is a metabolic disease that threatens human health. Tea is a healthy beverage with an abundance of benefits. This study revealed the uric acid-lowering efficacy of six types of tea water extracts (TWEs) on HUA in mice. The results revealed that under the intervention of TWEs, the expression of XDH, a key enzyme that produces uric acid, was significantly downregulated in the liver. TWE treatment significantly upregulated the expression of uric acid secretion transporters ABCG2, OAT1, and OAT3, and downregulated the expression of uric acid reabsorption transporter URAT1 in the kidney. Furthermore, HUA-induced oxidative stress could be alleviated by upregulating the Nrf2/HO-1 pathway. The intervention of TWEs also significantly upregulated the expression of the intestinal ABCG2 protein. On the other hand, TWE intervention could significantly upregulate the expression of intestinal ABCG2 and alleviate HUA by modulating the gut microbiota. Taken together, tea can comprehensively regulate uric acid metabolism in HUA mice. Interestingly, we found that the degree of fermentation of tea was negatively correlated with the uric acid-lowering effect. The current study indicated that tea consumption may have a mitigating effect on the HUA population and provided a basis for further research on the efficacy of tea on the dosage and mechanism of uric acid-lowering effects in humans.

## 1. Introduction

Hyperuricemia (HUA) refers to the fact that under a normal purine diet, the fasting serum uric acid level is higher than 420 μmol/L in men and 360 μmol/L in women [1]. Long-term high uric acid can lead to a series of diseases, including chronic kidney disease, insulin resistance, kidney stones, and gout, which has become a metabolic disease that threatens human health [2]. In recent years, the incidence of hyperuricemia has increased year by year, and it has gradually become a research hotspot in the medical field. At present, allopurinol, febuxostat, benzbromarone, etc. are currently used to treat hyperuricemia. These drugs have side effects such as uric acid stones, liver and kidney stones, and liver damage, which may not be tolerated by patients [3]. Therefore, it is particularly important to develop safe, non-toxic, and economically functional foods and nutraceuticals for replacement therapy.

Tea is the second most popular beverage in the world after water [4]. It has been documented for over 4000 years in China. According to the different processing methods and extent of fermentation, tea is divided into six types: unfermented green tea (GT), mildly fermented white tea (WT), lightly fermented yellow tea (YT), semi-fermented oolong tea (OT), fully fermented black tea (BT), and post-fermented dark tea (DT) [5]. Different fermentation processing techniques not only endow tea with rich flavors, but also the different biochemical reactions in the tea-making process make the ratio and composition of the ingredients subtly change, which allows different types of tea to have different health benefits [6]. These health benefits are derived from the complex content of the tea, such as tea polyphenols, theanine, tea polysaccharides, and caffeine, which have been reported to be good for health [7,8]. Studies have confirmed that tea has the effects of reducing lipids and weight loss, blood sugar, blood pressure, etc., to relieve metabolic syndrome [9], and it has also gradually attracted attention in reducing uric acid [10].

Hyperuricemia is caused by a disturbance of uric acid metabolism, a complex physiological process involving multiple organs (liver, kidney, and intestine). Uric acid is formed in the liver by purine dehydrogenase or oxidase degrading purines, and 2/3–3/4 of the total uric acid produced by the body every day is excreted through the kidneys in the urine, and the rest is excreted into the intestines through the hepatobiliary system [11]. At present, the research on tea in hyperuricemia is mostly limited to the research of single tea. Studies have reported that Pu-erh ripened tea inhibited the activities of xanthine oxidase and adenosine deaminase in the liver of hyperuricemia mice, and downregulated the mRNA expression levels of URAT1 and GLUT9 in the kidneys [12]. Zhu et al. showed that BT had a slightly better inhibitory effect on xanthine oxidase and adenosine deaminase activity than GT in hyper-uric acid mice [13]. Whether the different ways in which tea is processed affect the effect of regulating uric acid metabolism, and what its mechanism of action is, still need to be studied. Based on our previous study, six major teas were confirmed to have inhibitory effects on uric acid production in vitro, and we also found that the degree of fermentation may be proportional to the effect of lowering uric acid [14]. Therefore, in this study, we further verified the mechanism of the six major teas in relieving hyperuricemia in vivo. This study will provide guidance for healthy tea drinking and scientific tea selection for people with high uric acid, and provide new ideas and a theoretical basis for developing natural products from tea to relieve hyperuricemia.

## 2. Materials and Methods

### 2.1. Preparation of Tea Water Extracts (TWEs)

The six major teas were processed by the Tea Research Institute of Guangdong Academy of Agricultural Sciences using the uniform specifications of fresh leaves of Yinghong No. 9 tea tree variety, one bud and two leaves, in 2020. The six types of tea were soaked in boiled water (tea/water (*w*/*v*) = 1:20) for 30 min, which was repeated three times. The filtrate was collected by filtration, stored in a 10 cm Petri dish, frozen at −80 °C for an overnight treatment, and then freeze-dried in a freeze dryer (FD-1A-50, Biocool, Beijing, China) for 24 h. Finally, the tea water extracts were collected separately in moisture-proof bags and stored in a desiccator.

### 2.2. Animal Experiments

**Figure 1:**
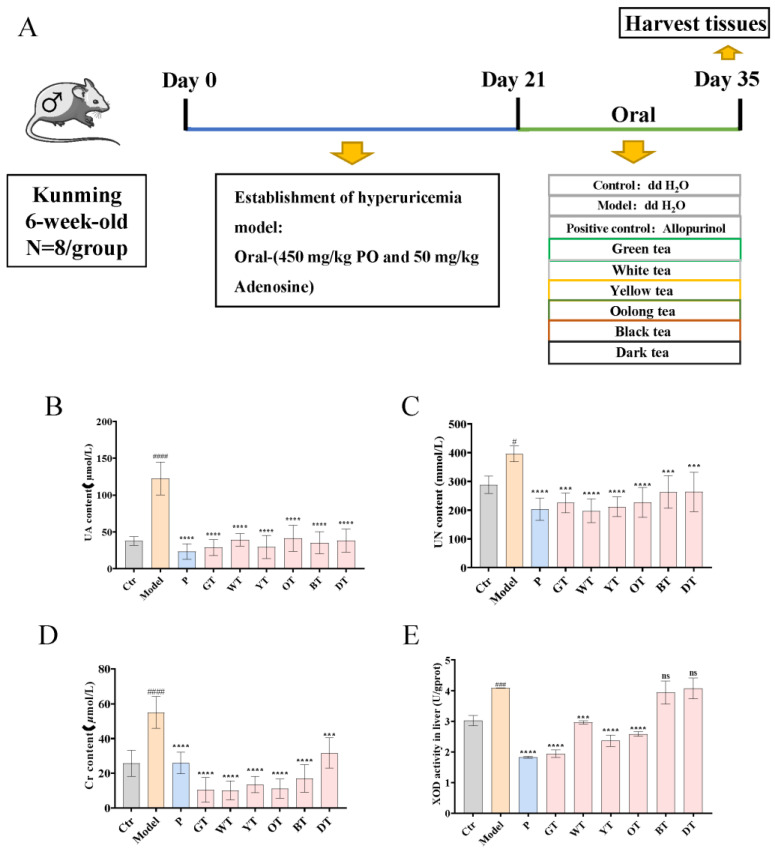
Effects of TWEs on UA-related indexes and antioxidant levels in HUA mice. (**A**) Study design for the whole experiment; (**B**) Serum UA levels. (**C**) Serum UN levels. (**D**) Serum Cr levels. (**E**) Liver XOD activity. The data are expressed as mean ± SD. ^#^ *p* < 0.1, ^###^ *p* < 0.001, ^####^ *p* < 0.0001 were compared with the control, ^ns^ *p* > 0.1, *** *p* < 0.001, **** *p* < 0.0001 were compared with the model.

All animal experiments were performed by the laboratory animal care and use guidelines and approved by the Animal Care and Welfare Committee of Tea Research Institute, Guangdong Academy of Agricultural Sciences (Serial Number: 2021004). Seventy-two male specific-pathogen-free (SPF) grade Kunming mice, with a weight of 35–40 g at 6 weeks of age, were purchased from the Guangzhou Chashi Ruihua Biotechnology Co., Ltd. (Lot: No: SHD-2021-07-05-15760). The mice were kept in a sterile laboratory maintained at a temperature of 24–25 °C and a humidity of 50–60% relative humidity on a standard 12 h light–dark cycle, with free access to water and food. After one week of acclimation, the mice were randomly divided into nine groups of eight mice each: (1) control, (0.5% CMC-Na); (2) model, potassium oxonate (450 mg/kg·d; S17112-25g, Yuanye Biological Technology, Shanghai, China) and adenosine (50 c; A4036-5G, Sigma-Aldrich, Shanghai, China); (3) allopurinol group: allopurinol (5 mg/kg·d; B27249, Yuanye Biological Technology, Shanghai, China); (4) green tea group (800 mg/kg·d); (5) white tea group (800 mg/kg·d); (6) yellow tea group (800 mg/kg·d); (7) oolong tea group (800 mg/kg·d); (8) black tea group (800 mg/kg·d); and (9) dark tea group (800 mg/kg·d). The TWE treatment dose was equivalent to an adult drinking 12–15 g of tea per day. The entire experimental period lasted 5 weeks. Except for the control group, the other groups were given potassium oxonate combined with adenosine by gavage, once a day, for three consecutive weeks, during which the control group was given 0.5% CNC-Na by gavage. Then, the allopurinol group and the six tea groups were respectively gavaged once a day for two consecutive weeks, during which the control group and the model were gavaged with 0.5% CNC-Na (Figure 1A). During the experiment, all mice had free access to food and water.

### 2.3. Biochemical Analysis

After 5 weeks of intervention, the mice were anesthetized with 40 mg/kg pentobarbital after a 12 h fast, and whole blood was obtained by cardiac puncture. Plasma was separated by centrifugation at 2500 rpm for 20 min and serum was stored at −80 °C. Liver, kidney, and small intestine tissues were collected, weighed, and photographed. Half of all tissues were stored at −80 °C and half were fixed in 10% neutral buffered formalin. According to the instructions (Nanjing Jiancheng Bioengineering Institute, Nanjing, China), commercially available kits were used to determine the changes in serum UA (C012-2-1), Cr (C011-2-1), UN (C013-2-1), GSH (A006-2-1), SOD (A001-3-2), and MDA (A003-1-2), as well as the changes in XOD (A002-1-1) activity in the liver (iMagic-V7 automatic analyzer, ICUBIO, Beijing, China). ROS levels were measured using MEIMIAN ELISA kits.

### 2.4. Calculation of Kidney Index

Based on the recordings of mouse kidneys and body weight, the kidney index was calculated as kidney mass (g)/body mass (g) × 100%.

### 2.5. Histopathological Evaluation

10% neutral buffered formalin-fixed liver, kidney, and small intestine tissues were dehydrated in ethanol gradients and embedded in paraffin. Then, 5 μm thick sections were cut, deparaffinized, rehydrated, stained with hematoxylin and eosin (H&E), and histopathological changes were observed under a light microscope (Olympus, Tokyo, Japan).

### 2.6. Immunohistochemistry

The fixed tissue was sectioned, dewaxed, and rehydrated, and sequentially soaked in double-distilled TBS for 2 min, TBS (Beijing Solarbio Science and Technology Co., Ltd., Beijing, China) for 5 min, and 3% H_2_O_2_ for 15 min in the dark to quench endogenous peroxidases. The sections were then rinsed with tap water, soaked again in TBS for 5 min, and microwaved in EDTA solution for antigen retrieval. After cooling to room temperature, the sections were washed thrice with TBS, demarcated with an oil pen, and blocked with 5% goat serum (Boster Biological Technology Co., Ltd., Beijing, China) for 30 min at room temperature. The blocking buffer was gently blotted out, and the sections were incubated overnight with suitable primary antibodies (diluted in blocking buffer; Boster, Wuhan, China) at 4 °C in a wet box. After washing thrice with TBS, the sections were incubated with secondary antibodies (Boster, Wuhan, China) at room temperature for 1 h, and washed again with TBS. The sections were then incubated with HRP-labeled streptavidin (Beyotime, Shanghai, China) for 1 h at room temperature and washed thrice with TBS, and the color was developed using a DAB reagent. After rinsing with tap water, the sections were counterstained with hematoxylin (Beyotime, Shanghai, China) for 1 min, and held under running tap water till all of the excess dye was washed out. Finally, the sections were dehydrated and sealed with neutral balsam (Beijing Solarbio Science and Technology Co. Ltd., Beijing, China), and observed under an Olympus BX-53 microscope (Olympus, Tokyo, Japan).

### 2.7. Western Blot Analysis

Proteins were extracted from liver tissues using a lysis buffer at 4 °C. The extracts were centrifuged at 13,200 rpm at 4 °C for 30 min, and the protein content of the supernatants was analyzed. Equal amounts of protein per sample were separated by 10% SDS-PAGE and transferred to PVDF membranes. The latter were blocked at room temperature with 5% skim milk in TBST for 1 h, and then incubated overnight with primary antibodies against XDH (Thr172), ABCG2, OAT1, OAT3, URAT1, Nrf2, HO-1 (Cell Signaling Technology, Danvers, MA, USA), and GAPDH (Sigma-Aldrich, St. Louis, MO, USA) at 4 °C. After washing thrice with TBST, the blots were incubated with secondary antibodies conjugated to horseradish peroxidase. The protein bands were visualized by enhanced chemiluminescence. The protein bands were grayscaled using ImageJ and the results were quantified compared to the loading control protein GAPDH.

### 2.8. High-Throughput Sequencing and Analysis of Fecal Microbiota

Fecal DNA was extracted using the E.Z.N.A. ^®^Stool DNA Kit (D4015, Omega, Inc., Albuquerque, NM, USA), labeled with specific barcodes, and the 16S fragment was amplified using specific primers. The amplified samples were sequenced on the Illumina NovaSeq platform and aligned against the 16S rRNA database using FLASH. High-quality QIIME2 was used to calculate alpha and beta diversity to determine species richness and diversity. UPGMA clustering was used to analyze the differences in the composition of gut microbiota at the phylum level. Moreover, further comparative analysis of the differences in the flora of the species composition in nine groups at the genus and species levels was conducted.

### 2.9. Statistical Analysis

All statistical analyses were conducted using Prism 8.0 software for Windows (GraphPad Software, La Jolla, CA, USA). Multiple groups were compared by analysis of variance (ANOVA) and Tukey’s post hoc test. *p*-values < 0.05 were considered statistically significant. The data are presented as the mean ± SD.

## 3. Results

### 3.1. TWEs Relieved Uric Acid Metabolism Disorder in HUA Mice

As shown in Figure 1, the UA, UN, and Cr levels of serum in the model group were significantly higher than those in the control group (Figure 1B–D), indicating that the HUA model was successfully established. In contrast, the mice in the allopurinol and TWEs groups had significantly lower serum levels of UA, UN, and Cr caused by HUA. In addition, we further detected the activity of XOD in the liver, with the results showing that the XOD activity was significantly increased in the model group, significantly decreased in allopurinol, green tea, white tea, yellow tea, and oolong tea groups, and showed no significant change in groups of black tea and dark tea (Figure 1E). It indicated that tea treatment could significantly alleviate uric acid production and excretion in HUA mice. Interestingly, lightly fermented tea may have a better effect than heavily fermented tea.

### 3.2. TWEs Reduced Hepatic Uric Acid Production in HUA Mice by Inhibiting XOD

**Figure 2:**
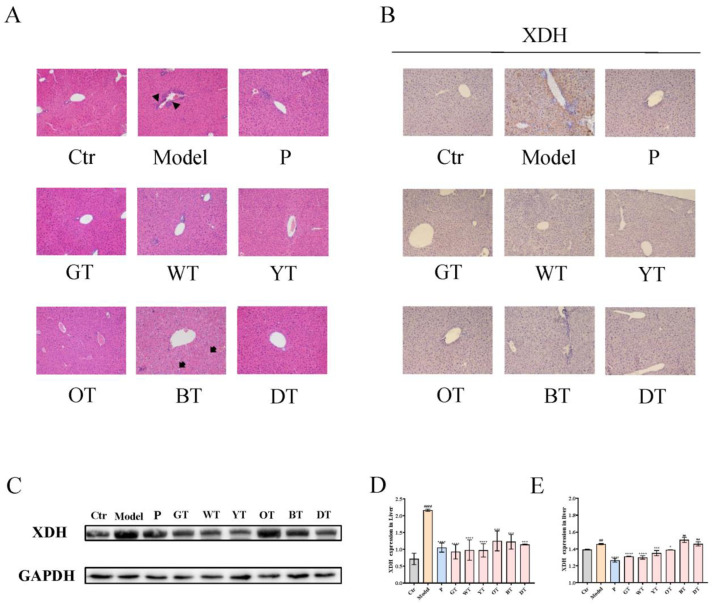
Mechanism of TWEs on UA metabolism in the liver. (**A**) Representative H&E-stained liver tissue images of different treatment groups: triangles indicate inflammatory infiltration and arrows indicate vacuolar degeneration of hepatocytes (200×). Representative IHC (**B**) and Western blotting (**C**) images of XDH expression in the liver. Quantification of XDH expression by Western blotting (**D**) and IHC (**E**). The data are expressed as mean ± SD. ^##^ *p* < 0.01, ^####^ *p* < 0.0001 were compared with the control, ^ns^ *p* > 0.1, * *p* < 0.1, *** *p* < 0.001, **** *p* < 0.0001 were compared with the model.

Through histopathological analysis, compared with the control group, the model group had mild inflammatory infiltration liver damage. Except for the black tea group, the liver tissue of the other groups recovered to the same situation as the control group after treatment, while the black tea group showed the phenomenon of vacuolar degeneration of hepatocytes (Figure 2A). XOD is a key enzyme in the production of uric acid. The IHC results showed that the XOD levels were downregulated in the model group. Treatment with allopurinol, green, white, yellow, and oolong tea water extracts significantly up-regulated the XOD levels. However, compared with the model group, there was no significant improvement after the treatment of black tea and black tea water extract (Figure 2B,E). On the other hand, the WB results indicated that the expression level of XOD was significantly upregulated in the liver of the model group, while it was significantly downregulated in the allopurinol and TWEs groups (Figure 2C,D). TWEs could reduce excess uric acid production by inhibiting XOD and had the potential to alleviate mild liver injury caused by HUA.

### 3.3. TWEs Alleviated HUA by Regulating Uric Acid Transporters in Renal

**Figure 3:**
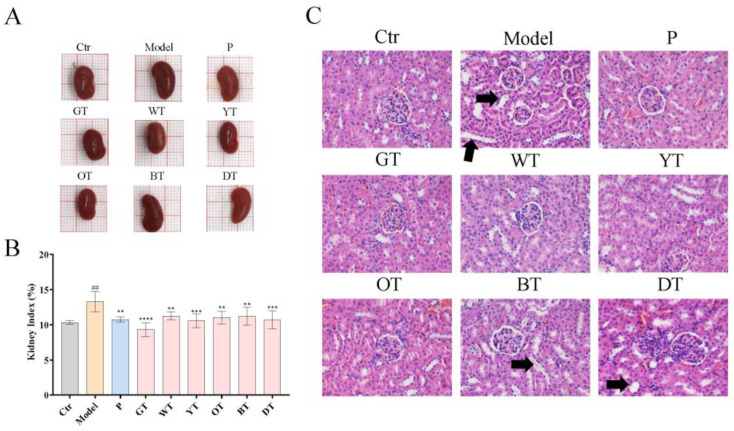
Six types of tea protected against kidney tissue damage in HUA mice. (**A**) Representative images of kidneys in each group. (**B**) Kidney index relative to body weight in the indicated groups. (**C**) Representative H&E-stained renal tissue images of different treatment groups, arrows indicated tubular dilation (200×). The data are expressed as mean ± SD. ^##^ *p* < 0.01 was compared with the control, ** *p* < 0.01, *** *p* < 0.001, **** *p* < 0.0001 were compared with the model.

The kidney is the key organ for the excretion of uric acid. As shown in Figure 3A,B, the kidney of the model group was significantly heavier compared to the control group. In contrast, treatment with TWEs significantly restored renal enlargement. In addition, the histopathological results showed that HUA caused significant tubular dilation and mild tubular epithelial cell necrosis in the model group. Under the intervention of TWEs, these symptoms were significantly improved in the green tea, white tea, and yellow tea groups, followed by the oolong tea group, and the black tea and dark tea groups had little effect.

**Figure 4:**
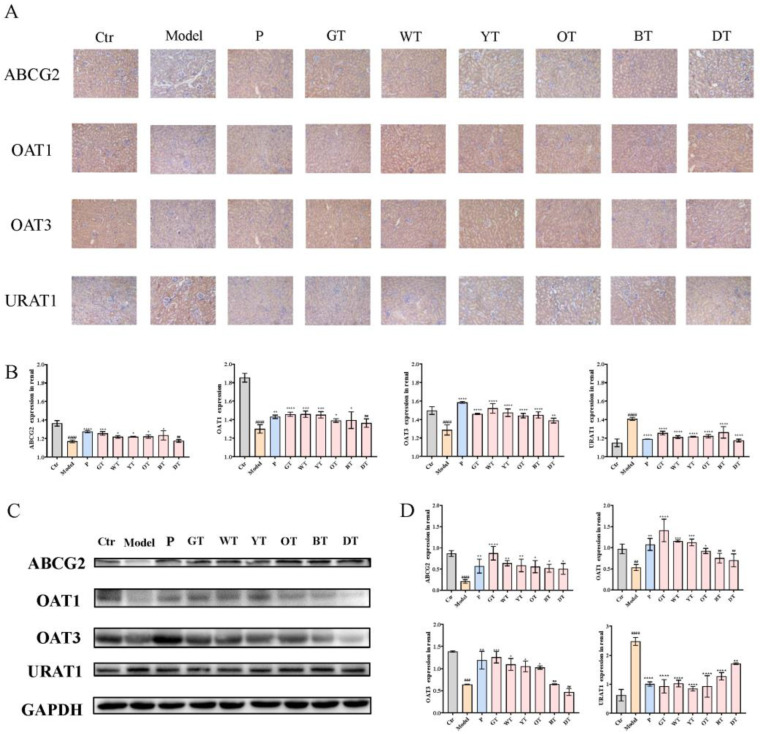
Mechanism of TWEs on UA metabolism in kidney. (**A**) Immunohistochemical staining of uric acid transporters in the kidney (200×). (**B**) T bar graphs show the positive rates of ABCG2, OAT1, OAT3, and URAT1 proteins in the kidney. (**C**) Representative immunoblots of ABCG2, OAT1, OAT3, and URAT1 protein expression in the kidney. (**D**) Quantification of ABCG2, OAT1, OAT3, and URAT1 expression levels. The data are expressed as mean ± SD. ^##^ *p* <0.01, ^###^ *p* < 0.001, ^####^ *p* < 0.0001 were compared with the control, ^ns^ *p* > 0.1, * *p* < 0.1, ** *p* < 0.01, *** *p* < 0.001, **** *p* < 0.0001 were compared with the model.

To explore the effects of TWEs on regulating renal uric acid transporters, we analyzed the expression of uric acid secretion transporters ABCG2, OAT1, OAT3, and uric acid reabsorption transporter URAT1. As shown in Figure 4, treatment with TWEs significantly upregulated ABCG2, OAT1, and OAT3, and downregulated the renal levels of URAT1.

### 3.4. TWEs Alleviated HUA-Induced Renal Injury by Inhibiting Oxidative Stress via Nrf2/HO-1 Pathway

**Figure 5:**
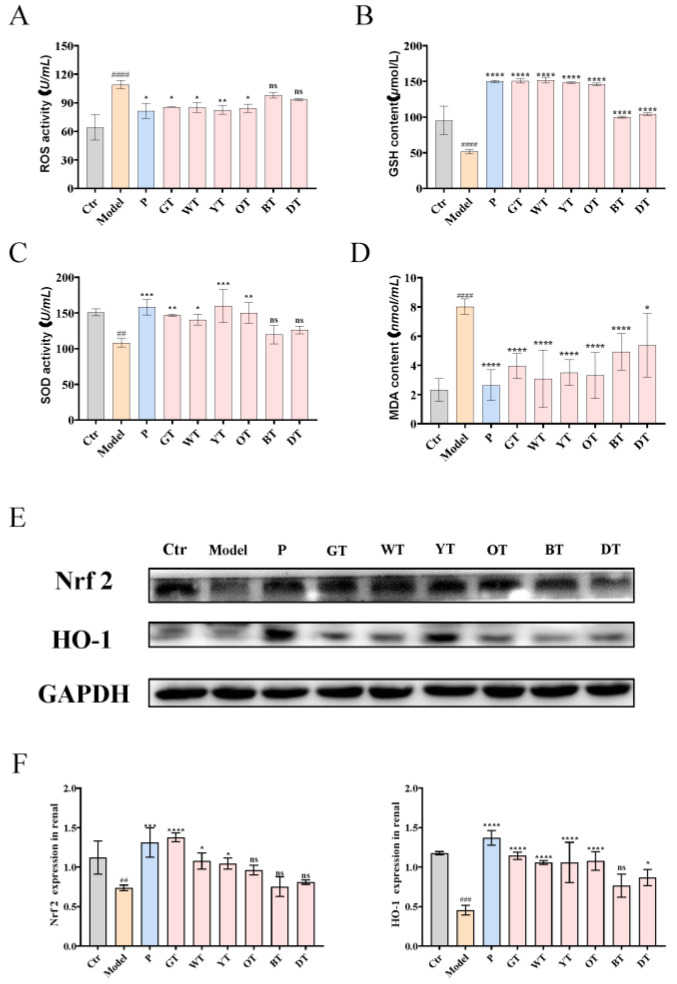
Mechanisms of TWEs in relieving renal oxidative stress. (**A**) Serum ROS activity. (**B**) Serum GSH levels. (**C**) Serum SOD levels. (**D**) Serum MDA activity. Representative immunoblots (**E**) images of Nrf2 and HO-1 expression in the kidney. Quantification of Nrf2 and HO-1 expression by Western blotting (**F**). The data are expressed as mean ± SD. ^##^ *p* < 0.01, ^###^ *p* < 0.001, ^####^ *p* < 0.0001 were compared with the control, ^ns^ *p* > 0.1, * *p* < 0.1, ** *p* <0.01, *** *p* < 0.001, **** *p* < 0.0001 were compared with the model.

To further verify the mechanism of TWEs alleviating HUA-induced renal injury, we detected the serum ROS level in mice and simultaneously detected the activity levels of GSH, MDA, and SOD. The results showed that compared with the control group, the model group produced more significant oxidative stress, and TWE treatment significantly alleviated oxidative stress in mice (Figure 5A–D). To further evaluate the effect of TWEs in alleviating oxidative stress, WB was used to detect the protein expression levels of Nrf2 and HO-1 in mouse renal tissue (Figure 5E,F). The data showed that the expression levels of Nrf2 and HO-1 in the model group were significantly lower than those in the control group. However, the levels of these proteins were significantly increased in mice treated with TWEs compared with the model group.

### 3.5. TWEs Promoted Intestinal Uric Acid Excretion by Upregulating ABCG2

**Figure 6:**
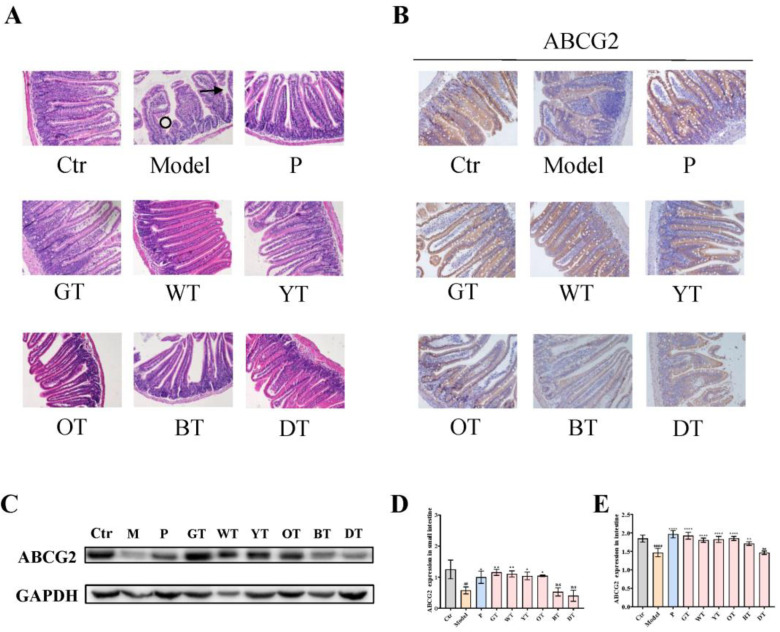
Mechanism of TWEs on UA metabolism in the small intestine. (**A**) Representative H&E-stained small intestine tissue images of different treatment groups: arrows indicate villous shedding and circles indicate abnormal crypt structure (200×). Representative immunoblots (**B**) and IHC (**C**) images of ABCG2 expression in the small intestine. Quantification of ABCG2 expression by Western blotting (**D**) and IHC (**E**). The data are expressed as mean ± SD. ^##^ *p* < 0.01, ^####^ *p* < 0.0001 were compared with the control, ^ns^ *p* > 0.1, * *p* < 0.1, ** *p* < 0.001, **** *p* < 0.0001 were compared with the model.

The intestine is one of the important organs for uric acid excretion. ABCG2 regulates intestinal uric acid excretion, thereby maintaining the balance of uric acid metabolism. Histopathological analysis of the small intestine showed that compared with the control group, the model group exhibited shedding of villi and abnormal crypt structure, which were alleviated by TWE treatment (Figure 6A). Then, we analyzed the expression level of uric acid secretion transporters ABCG2, as shown in Figure 6B–E, and the results revealed that compared with untreated normal mice, the expression levels of ABCG2 in the intestines of TWEs-treated mice were all elevated. Among them, except for the dark tea group, all showed significant effects. Overall, compared with heavily fermented tea, lightly fermented tea may have a more significant promoting effect on the expression of intestinal uric acid secretion transporter ABCG2 in general.

### 3.6. TWEs Alleviated Uric Acid Metabolism Disorders by Regulating Gut Microbiota

**Figure 7:**
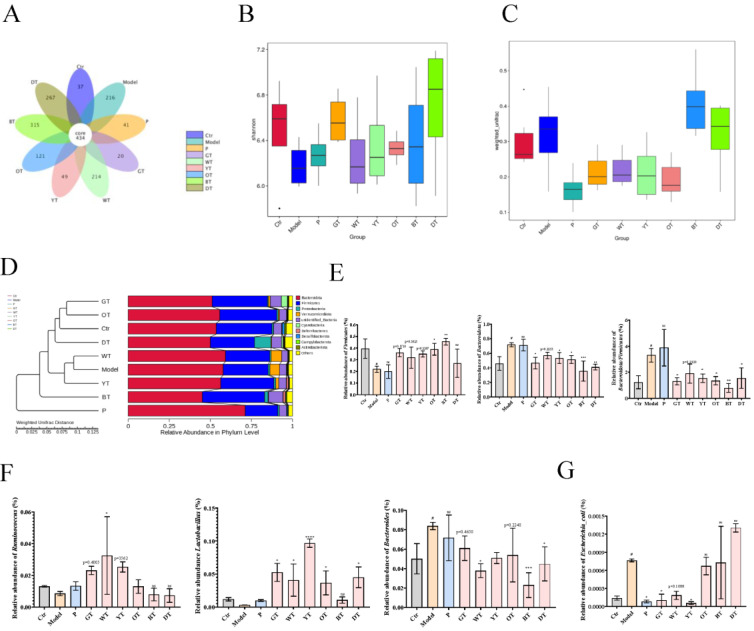
Effects of TWEs on gut microbiota in HUA mice. (**A**) Venn diagram showing species composition in nine groups. (**B**) Shannon index analysis of alpha diversity of nine taxa species’ intergroup difference analysis. (**C**) Boxplots based on weighted UniFrac beta diversity of nine taxa species. (**D**) Weighted UniFrac distance-based UPGMA clustering tree for nine taxa species at the phylum level. (**E**) Relative abundance of *Firmicutes*, *Bacteroidota*, and *Firmicutes*/*Bacteroidota* in nine taxa species at the phylum level. (**F**) Relative abundance of *Ruminococcus*, *Lactobacillus*, and *Bacteroides* in nine taxa species at the genus level. (**G**) Relative abundance of *Escherichia coli* in nine taxa species at the genus level. The data are expressed as mean ± SD. ^#^ *p* < 0. 1 was compared with the control, ^ns^ *p* > 0.1, * *p* < 0.1, ** *p* < 0.001, *** *p* < 0.001, **** *p* < 0.0001 were compared with the model.

By sequencing the 16S rRNA V3-V4 region of fecal DNA extracted from different groups of mice (Eight mice per group), according to species annotation, the alpha diversity and beta diversity were further calculated, and the differences between groups were compared and analyzed. This revealed the effect of TWE treatment on the HUA structure of the mouse gut microbial community. As shown in Figure 7A, the specific OTU numbers of intestinal flora in the control group, model group, positive drug group, GT group, WT group, YT group, OT group, BT group, and DT group were 37, 216, 41, 20, 214, 49, 121, 315, and 267, respectively, which showed that the species composition of the fecal microbiota of HUA mice and different TWE treatments were changed. Changes in gut microbial communities were further assessed in terms of alpha and beta diversity. The combined results of Shannon’s analysis of alpha diversity and boxplots based on weighted UniFrac beta diversity showed that there were differences in species diversity among different taxa, and the diversity and richness of the fecal microbiota of mice treated with TWEs showed signs of recovery. Compared with the control group, the species composition of the model group was more abundant; the DT group had the most abundance of species composition diversity in the fecal microbiota of mice treated with TWEs, followed by the GT group, which also showed a rich species diversity (Figure 7B,C). The results of the phylum-level UPGMA clustering tree analysis based on weighted UniFrac distances showed that the relative abundance of *Firmicutes* decreased significantly in the model group and increased after TWE treatment, while the relative abundance of *Bacteroidota* increased significantly and decreased after TWE treatment. In addition, the relative abundance of the *Firmicutes*/*Bacteroidota* ratio increased significantly in the model group, but decreased after TWE treatment, indicating that TWE treatment had a certain alleviating effect on HUA (Figure 7D,E). Furthermore, through the relative abundance analysis of *Ruminococcus*, *Lactobacillus*, and *Bacteroides* at the genus level, the results showed that TWE treatment promoted the metabolism of uric acid, and alleviated HUA by upregulating the relative abundance of *Ruminococcus* and *Lactobacillus*. On the other hand, TWE treatment reduced the relative abundance of *Bacteroides*, inhibited the uric acid synthesis and alleviated HUA (Figure 7F). Moreover, the species-level results indicated that TWE treatment reduced the relative abundance of *Escherichia coli*, inhibited the secretion of XOD, reduced uric acid production, and thereby alleviated HUA (Figure 7G).

## 4. Discussion

As one of the most popular natural botanical beverages today, tea has been shown to benefit human health, such as lowering uric acid [15]. The same tea leaves can be made into different types of tea according to different processing methods. The fermentation process has a significant impact on the composition of tea leaves, thus endowing the tea with different health benefits [6]. Research has found that for carbon tetrachloride-induced liver damage in mice, heavily fermented teas relieved liver damage through its anti-inflammatory properties, while lightly fermented teas mainly relieved liver damage by inhibiting the oxidative stress [16]. In addition, the study found that compared with green tea, fully fermented tea had a more significant inhibitory effect on the formation of β-amyloid and Aβ42 mediated by age-related dyslipidemia [17]. With the improvement in people’s living conditions, the incidence of hyperuricemia is increasing year by year. The incidence of hyperuricemia in different ethnic groups in the world is 2.6% to 36%, and the incidence in mainland China is 13.3% (male: 19.4%, female: 7.9%) [18], making HUA an urgent problem to be solved in the medical field. In our previous study in vitro, the degree of fermentation was found to be positively correlated with the inhibition of XOD activity by tea [14]. Therefore, we further explored the reduction of uric acid production effect and mechanism in vivo using six types of tea processed from the same tea leaves. First, we established a hyperuricemia mouse model by continuous gavage of 450 mg/kg potassium oxonate and 50 mg/kg adenosine for three weeks. The model was established by improvements based on previous experiments [19,20]. Subsequently, we orally treated hyperuricemia mice with TWEs at a dose of 800 mg/kg for 2 weeks (the equivalent of an adult drinking 12–15 g of tea a day). In reported studies, the commonly measured concentration range of TWEs treatment in mouse animal models is 500 to 1500 mg/kg per day [6,12]. This concentration range has essentially no other toxicity or side effects in mice [13]. Since we already had six teas as experimental treatment groups in this study, we did not conduct experimental treatment studies with different doses of the same tea. We will continue to investigate the optimal treatment dose and the main active components of the uric acid-lowering effect of tea in future studies. In addition, tea, as a class of functional beverages, has the potential to replace the uric acid-lowering efficacy of drugs. A study found that green tea catechins can enhance the metabolism of uric acid in men after drinking [21]. Not only that, green tea extract has also been reported to have a uric acid-lowering effect on patients with hyperuricemia [22]. Therefore, further experiments are needed to verify the mechanism and main components of the effect of tea on the alleviation of hyperuricemia in humans.

The liver is the most critical organ for uric acid synthesis, and excess uric acid production will cause metabolic disorders. XOD is a key enzyme in the production of uric acid in the liver, which can catalyze hypoxanthine to xanthine and, finally, uric acid [23]. The research found that Lycium barbarum polysaccharides, salvia plebeia extract, and soy sauce could relieve HUA by inhibiting XOD [24,25,26]. In this study, we found that TWEs can downregulate the expression level of XOD. It was also interesting to find that the degree of fermentation was negatively correlated with it, which was consistent with our previous study. On the other hand, the efficacy of tea in alleviating HUA by inhibiting XOD is mostly limited to the research on green tea. Moreover, the abundant polyphenols in green tea and their main constituent catechins have also been recorded to inhibit XOD activity [27,28]. In addition, black tea has also been studied, which may have a better XOD inhibitory effect than green tea [13], which is inconsistent with our conclusions. The materials they used were not derived from the same tea species, while the influence of tea tree varieties on the content of functional components in tea cannot be ignored [29]. In this study, we used the same tea species to process six major tea types, which better avoided the influence of tea species differences on the experiment, which made our conclusions more credible. Taken together, we innovatively revealed in this study that tea can inhibit the production of uric acid by inhibiting XOD at the animal level, and it was negatively correlated with the degree of fermentation.

The kidney is the main organ for the excretion of uric acid. About 75% of the uric acid in the body is excreted by the kidneys and finally excreted in the form of urine [20]. Uric acid metabolism is regulated by secretion and reabsorption transporters. Among the secreted proteins, the dysfunction of ABCG2 (an ATP-driven efflux pump) has become one of the major factors in human hyperuricemia; OAT1 (encoded by SLC22A6)/OAT3 (encoded by SLC22A8) driven by α-KG leads to uric acid being secreted from the basolateral side of the cells to the renal tubular cells, and finally excreted in the urine. Uric acid transporters coordinate with each other in structure, function, and location to regulate the balance of uric acid in the human body [23]. The uric acid reabsorption transporter URAT1 encoded by the hyperuricemia-related gene SLC22A12 reabsorbs urate from filtered urine [20]. This study clarified that under the action of TWEs, the expressions of ABCG2, OAT1, and OAT3 were significantly upregulated, and the expression of URAT1 was significantly downregulated in HUA mice. The mechanism of TWEs regulating transporters to relieve HUA followed the laws of uric acid metabolism. Apart from this, hyperuricemia was found can mediate mitochondrial calcium overload and ultimately lead to endothelial dysfunction through mitochondrial Na^+^/Ca^2+^ exchange, thereby increasing ROS [30]. Nrf2 was an oxidative stress sensor and key transcription factor that protected cells from foreign bodies and oxidative damage. When ROS levels were elevated, Nrf2 enhanced the transcription of anti-oxidative stress proteins including HO-1 [31]. Here, we found that TWEs can significantly alleviate HUA-induced ROS through the Nrf2/HO-1 signaling pathway. Previous studies have revealed that polyphenols in tea include monomeric EGCG, and the polyphenol oxidation product theaflavins can alleviate oxidative stress injury through the Nrf2/HO-1 pathway [32,33,34]. This provided a strong basis for the reliability of our conclusions. Intriguingly, here, we found the same regularity as in the liver: the fermentation time was negatively correlated with the uric acid-lowering efficacy of the TWEs. Similarly, the fermentation time was negatively correlated with TWEs’ alleviation of oxidative stress damage. With the increase in fermentation time, the polyphenols in tea were gradually converted into polyphenol oxides. In our previous in vitro cell experiments, it was found that the inhibitory effect of polyphenols catechins on XOD was better than that of polyphenol oxide theaflavins, and the higher polyphenol content in mildly fermented tea was significantly better than that of caffeine and theabrownin in heavily fermented tea [14]. This may be one of the reasons why the fermentation time was negatively correlated with the uric acid-lowering efficacy of the TWEs.

The gut also plays an important role in the excretion of uric acid, with about a quarter of uric acid being excreted into the gut. As the main site of digestion and absorption, the small intestine is also the main site of uric acid metabolism in the intestinal tract [35]. ABCG2 is the main transporter of uric acid excretion in the intestine [20]. Arshad Mehmood et al. reported that the upregulation of ABCG2 can alleviate HUA [35]. Consistent with our conclusions, significant upregulation of ABCG2 expression in the small intestine alleviated HUA under TWE intervention. In the small intestinal tissue, the degree of TWE fermentation was still negatively correlated with the HUA-relieving effect, which was consistent with the above conclusions. On the other hand, as awareness of the impact of gut microbiota on health continues to increase, the gut microbiota has been identified as a new target for the treatment of HUA [36]. The intervention of tea on intestinal flora has received extensive attention. Tea has a similar prebiotic effect, and the most significant effect is to reduce the imbalance of the intestinal flora caused by a high-fat diet. Among them, green tea restored the ratio of Firmicutes to Bacteroidetes in the gut of mice fed a high-fat diet. Black tea can increase the proportion of gut bacteria called *Pseudobutyrivibrio* in the gut microbiota, which affects energy metabolism in the liver and can help people lose weight, and this effect seems to be better than green tea. Dark tea such as Pu-erh can significantly reduce the number of bacteria containing bile salt hydrolase in the gut, dietary fat, and the functional activity of bile salt hydrolase to reduce obesity [37,38,39]. Our conclusions showed that TWE treatment modulated the relative abundance of *Lactobacillus* and *Escherichia coli* in the gut of HUA mice, thereby regulating intestinal uric acid production and metabolism, which was consistent with the conclusion of Wangle et al., who found that gut microbes promoted the catabolism of purine, and the excretion of uric acids such as *Escherichia coli* can produce the enzyme responsible for the oxidative metabolism of purine xanthine dehydrogenase. *Lactobacillus* and *Pseudomonas* in the intestinal tract can decompose uric acid transported to the intestinal lumen. [40]. In addition, Yiran Yu et al. found that *Ruminococcus* and *Lactobacillus* were decreased in the HUA rat model, which provided a scientific basis for our conclusion. We detected the gut microbiota at the mouse genus level and found that *Ruminococcus* and *Lactobacillus* in the HUA mouse model decreased, while the relative abundance of the two microbiota was restored after TWE treatment [36]. On the other hand, studies have reported the overgrowth of pathogenic bacteria *Escherichia coli* and *Bacteroidetes* in HUA rats [41], which was consistent with our conclusion that these conditions were alleviated after treatment with TWEs. In summary, under the intervention of TWEs, HUA can be alleviated by regulating the uric acid transporter ABCG2 in HUA mice. Not only that, but TWEs may also regulate uric acid metabolism by regulating the composition of gut microbiota. Taken together, long-term regular drinking of unfermented or lightly fermented tea can alleviate HUA.

## 5. Conclusions

In this study, TWEs were found to alleviate HUA in several ways: (1) downregulation of XDH protein expression to reduce hepatic uric acid production; (2) regulating uric acid transporter to promote renal uric acid excretion and relieve HUA-induced oxidative stress through the Nrf2/HO-1 pathway; (3) upregulating the expression level of ABCG2 to promote intestinal uric acid excretion, and may also regulate the intestinal flora to relieve HUA. Furthermore, unfermented and lightly fermented teas were generally more potent than more fermented teas. Thus, long-term regular tea consumption may have the potential to alleviate HUA, but its optimal dose and effects on humans need further experimental validation.

## Data Availability

The data presented in this study are available on request from the corresponding author. The data are not publicly available due to privacy reasons.

## Abbreviations

HUA	hyperuricemia
TWEs	tea water extracts
WB	Western blot
IHC	immunohistochemical
URAT1	urate anion transporter 1
GLUT9	glucose transporter 9
OAT1	organic anion transporters 1
OAT3	organic anion transporters 3
ABCG2	ATP-binding cassette sub-family G member 2
XOD	xanthine oxidase
XDH	xanthine dehydrogenase
Nrf2	nuclear factor erythroid2-related factor 2
HO-1	Heme Oxygenase 1
ROS	reactive oxygen species
GSH	reduced glutathione
MDA	malondialdehyde
SOD	superoxide dismutase
UA	uric acid
UN	urea nitrogen
Cr	creatine
CMC-Na	carboxymethylcellulose sodium
BW	body weight
UPGMA	unweighted pair-group method with arithmetic means.
